# Human‐in‐the‐loop optimized rocker profile of running shoes to enhance ankle work and running economy

**DOI:** 10.1002/ejsc.12054

**Published:** 2024-01-30

**Authors:** Thijs Tankink, Han Houdijk, Juha M. Hijmans

**Affiliations:** ^1^ Department of Human Movement Sciences University of Groningen University Medical Center Groningen Groningen The Netherlands; ^2^ Department of Rehabilitation Medicine University of Groningen University Medical Center Groningen Groningen The Netherlands

**Keywords:** ankle gearing, biomechanics, metabolic rate, rocker shoes, Triceps Surae

## Abstract

Increasing the efficiency at which muscles generate mechanical power could improve running economy. A potential way to reduce muscle fiber shortening velocities and enhance energy storing of the Triceps Surae is changing their gear ratio at the ankle via optimization of shoe rollover profile. The aim of the current study was to individually optimize rollover profile of rocker shoes via human‐in‐the‐loop optimization to maximize positive ankle work to redistribute joint work from the hip and knee to the ankle and improve running economy. A total of 10 runners ran on a treadmill with experimental rocker shoes in which apex position and angle were optimized using an evolution algorithm to maximize positive ankle work. We compared experimental shoes with optimal settings, standard settings, and control shoes in terms of biomechanics and running economy. Optimal apex parameters differed considerably between participants. The optimal condition resulted in higher positive ankle work and a higher proportional share of the ankle in the total positive lower limb work compared to the standard condition. A difference in running economy between these conditions was not found. Human‐in‐the‐loop optimization can redistribute joint work from the hip and knee to the ankle by individually optimizing apex parameters. Although this did not improve running economy, the study showed that human‐in‐the‐loop optimization could improve the effectiveness of footwear with respect to the selected optimization parameter on an individual level.

## Highlights

- Human‐in‐the‐loop optimization is able to individually optimize apex position and apex angle of running shoes to enhance ankle work
- The increase in positive ankle work results in a redistribution of positive work generated around the lower limb joints from the hip and knee to the ankle
- The increase ankle work did not result in an increased running economy and therefore it should be further investigated which factors confound the theoretical benefit

## 1 INTRODUCTION

Since the marathon distance (42.195 km) was standardized, record times have progressively improved (Joyner et al., 2011). Despite that, these improvements extrapolate to a sub‐2‐h performance in the near future, some scientists and athletes considered this as physiologically impossible (Hill, 1925; Weiss et al., 2016). However, the discussion is more topical than ever, after Eliud Kipchoge recently improved the official world record in 2:01:09 in the 2022 Berlin Marathon and broke the 2‐h barrier using pacers and drafting. These achievements were partly assigned to the newly developed running shoes that were worn (Hoogkamer et al., 2018).

Athletes, manufacturers, and scientists have investigated several shoe modifications to improve running performance, especially in terms of running economy (Moore, 2016). Running economy partially depends on the efficiency in which muscles generate mechanical power (Cavagna et al., 1977). This is dependent on the built‐in force‐velocity and force–length properties of skeletal muscles (Hill, 1938), where slower shortening velocities of muscle fibers and working close to their optimal length results in more efficient production of force and mechanical work (Fenn et al., 1935). Another important factor contributing to running economy is the spring‐like behavior of the human leg. During the first half of ground contact, the leg compresses while negative power is performed on the body's center of mass, and in the second half, the leg extends again while positive power is produced. Part of the negative mechanical power can be stored in tendons and other tissues around the lower limb joints, and when returned reducing the need to actively generate power in muscle fibers (Cavanagh et al., 1977).

Especially, the Triceps Surae contain morphologies that are suitable for these processes because a considerable part of the change of muscle tendon unit can be taken up by stretch and recoil of their long compliant series elastic elements (Ishikawa et al., 2007), limiting muscle fiber contraction velocities (Roberts, 2002). More proximal leg muscles do have these characteristics to a lesser extent (Ker et al., 2021), and therefore their change in length is mostly a consequence of changes in muscle fiber length. This results in energy loss during eccentric contraction and higher shortening velocities during concentric contraction, which makes the contractile conditions of the proximal leg muscles less efficient compared to the distal leg muscles. A previous study already suggested that a redistribution of joint work from the ankle to the knee and hip could contribute to a reduced running economy (Sanno et al., 2018). Moreover, runners with higher running economy generally have a higher energy storage capacity in the Triceps Surae compared to runners with lower running economy (Arampatzis et al., 2006).

One way to enhance energy storing in the tendon of the ankle plantar flexor muscles and reduce muscle fiber shortening velocity is to change the gear ratio at the ankle (Carrier et al., 1994; Ray et al., 2020). This is the ratio of the lever arm of the ground reaction force and the lever arm of the Triceps Surae with respect to the ankle joint. Increasing the gear ratio without changing the magnitude of the ground reaction force would demand a greater force from the calf muscles to generate the required muscle moment. This increased muscle force increases the stretch in the tendon and therewith reduces the required lengthening and shortening of the muscle fiber at a given ankle angular change. Increasing the gear ratio can be caused by a more distally placed apex position of the shoe compared to the metatarsophalangeal (MTP) joint, which causes a distal displacement of the point of application of the ground reaction force (Sobhani et al., 2013), with an increased ankle moment and with the same angular velocity increased positive ankle power.

In addition, the apex angle of a running shoe can affect the gear ratio. Push‐off can be performed around different axes: an axis through the MTP joint of the first and second toe (MTP_1‐2_), corresponding with an apex angle of about 90°, and an axis through the MTP joint of the second to the fifth toe (MTP_2‐5_) with an apex angle of 50˚–70° (Bojsen‐Møller, 1978). These 2 push‐off mechanisms result in moment arms with different magnitudes. The ratio between the arms (MTP_1‐2_:MTP_2‐5_) is 1.22:1 (Bojsen‐Møller, 1978). Thus, it is expected that an apex angle of about 90° is beneficial for the calf muscle efficiency through increased gearing.

Most previous studies (Ray et al., 2020; Roy et al., 2006) typically investigate only 1 single shoe parameter out of many that are relevant. This limits the exploration of the interaction between these parameters. To complicate the challenge, physiological and neurological differences between individuals can cause different responses to the same intervention (Logan et al., 2010), and responses can change substantially during the course of adaptation (Hollander et al., 2019).

A potential method to overcome these challenges is human‐in‐the‐loop optimization (Zhang et al., 2017), in which the human being is included ‘in vivo’ in the control loop and device parameters are systematically varied during the measurement period using an intelligent optimization algorithm in response to measured performance to optimize human performance. By means of this method, the parameters could be tuned to the needs of the individual (Scotto di Luzio et al., 2018) while taking into account the natural behavior of humans to simultaneously optimize coordination patterns with respect to locomotor performance (Alexander, 1989). Frequently, metabolic cost is used directly as an objective for optimization (Zhang et al., 2017). However, this requires relatively long measurements, as steady state needs to be reached, which could lead to fatigue when used for running. Mechanical work at the ankle, which requires less measurement time to determine and could enhance running economy via the morphologies of the Triceps Surae as described above, could potentially be used as a proxy for metabolic cost.

Therefore, the aim of the present study is to individually optimize the apex position and apex angle of running shoes through human‐in‐the‐loop optimization to maximize positive ankle work, generated during steady state running. We hypothesize that optimization will result in a more distally placed apex position compared to a normal running shoe and an apex angle close to 90°. We expect that the increase in positive ankle work causes a higher proportional share of positive ankle work to the total positive work generated around the lower limb joints (hip, knee, and ankle), which will result in an improved running economy.

## 2 METHOD

### 2.1 Participants

A total of 10 (2 female) recreational runners (age: 22.0 ± 1.8 years; height: 183.0 ± 8.5 cm, weight: 77.7 ± 11.1 kg, shoe size range: 38–44 EU) participated in this study. Participants had to run at least 15 km per week to be included. Their running distances ranged from 15 to 80 km per week (26.7 ± 20.6 km/week) as part of regular training. All participants were free of injury and pain at the time of the experiment and signed an informed consent. The study protocol was approved by the local ethics committee of the department of Human Movement Sciences, University Medical Center Groningen (10349).

### 2.2 Shoe conditions

**FIGURE 1:**
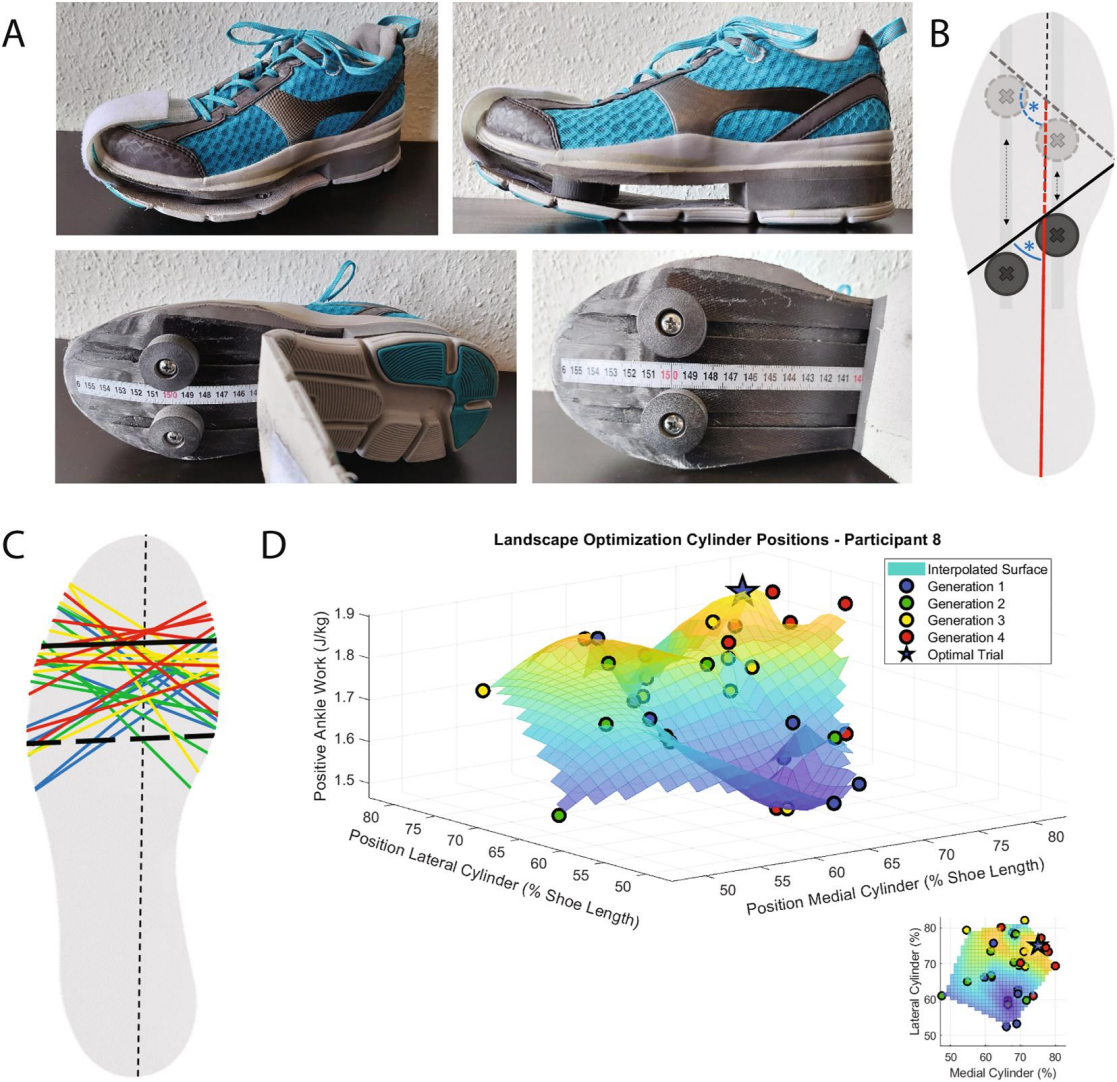
(A) Experimental shoes with adjustable apex position and apex angle. The position of the cylinders (bottom) could be changed across the rails, by manually screwing the bolts. (B) Changing the cylinders result in a new (dashed) apex position (red) and apex angle (blue asterisk). (C + D) Example of the optimization results for 1 participant. (C) The apex line of every trial is presented. The colors represent the trials of a given generation as indicated in the legend on the right. The black lines indicate the optimal settings (solid) and standard settings (dashed). (D) Positive ankle work (vertical axis) as a function of the cylinder positions (medial and lateral; as percentage of total shoe length) of every trial. A small 2D top view is inserted in the right bottom corner. The different colored circles indicate the eight trials of the different generations. The optimal trial is represented by the star.

We used regular athletic shoes (Chris, Dr Comfort, Mequon, WI, USA) as a control condition and experimental shoes (Figure 1A) with an adjustable apex position and apex angle (Reints, 2020) made from the same shoe model. The outer sole of the experimental shoes was removed and 3 carbon fiber layers that contained 2 rails with sliders were placed below the shoe, which could be used to manipulate the apex position and angle of the sole (Figure 1B). The carbon plates resulted in a higher stiffness (83.6 N/rad) and mass (range: 407–560 g/shoe) compared to the control shoe (1.9 N/rad; mass range: 197–261 g/shoe). Two 3D‐printed cylinders (diameter = 30 mm; height = 13 mm) with tapered rims were screwed onto the sliders with a bolt. The position of the cylinders could be changed across the rails (40%–90% total shoe length) by manually screwing the bolts. A polyethylene layer of 3 mm (Streifylast, Streifeneder, Emmering, Germany) was placed between the original outer sole and the cylinders to prevent the outer sole being damaged.

### 2.3 Experimental set‐up

Measurements were performed using the GRAIL (Gait Realtime Analysis Interactive Lab; Motekforce Link, Amsterdam, the Netherlands) in the department of Human Movement Sciences of the University Medical Center Groningen. The GRAIL consists of a dual‐belt treadmill with force sensors beneath each separate belt (Motekforce Link, Amsterdam, the Netherlands; Fs = 900 Hz) and a ten‐camera (Vero) motion capture system (Vicon Nexus 2.8.1, Oxford, UK; Fs = 150 Hz) that registered the marker trajectories of 22 reflective markers (diameter = 14 mm) placed according to the lower‐limb Human Body Model 2 (van den Bo et al., 2013). We used the D‐flow (3.34.3, Motekforce Link, the Netherlands, Amsterdam) online environment for real‐time analysis during the optimization period. Respiratory data were collected in the evaluation trials by the portable metabolic analyzer K5 (COSMED, Rome, Italy) in breath‐by‐breath mode.

### 2.4 Experimental protocol

The experiment was performed during 1 lab visit and consisted of a warming up, an optimization period, and an evaluation phase. The protocol started with a warming up of 5 min while wearing the experimental shoes with an apex position of 65% total shoe length (middle limits cylinder positions) and an apex angle of 90°. During the warming up, participants chose a self‐selected sub‐maximal running speed. To minimize the influence of fatigue, participants were instructed to select a speed that they could keep up for 60 min and had a 60‐s rest break after every running trial. The self‐selected speed (2.67 ± 0.44 m/s) was maintained during the rest of the protocol.

During the optimization period, we aimed to maximize the positive ankle work of the dominant leg (foot used to kick a ball) for every individual participant by optimizing the apex position and apex angle. These parameters were manually adapted by screwing the bolts and changing the cylinder positions of both the left and right shoe during the rest periods. We used a covariance matrix adaptation evolution strategy (CMA‐ES) (Hansen, 2006) to optimize the apex position and angle. The algorithm used a predetermined number (8) of measurements, forming 1 generation, to calculate the parameter values of the next generation. After every generation, the apex parameters of the 4 trials that resulted in the highest positive ankle work were selected as input for the algorithm to generate 8 new candidates for the parameter values of the following generation. These values are around the mean parameters of these 4 best trials. With every new generation, the value of this mean is updated and the area of search around this mean was further reduced to converge to the optimal apex parameter and angle. Based on previous studies (Hansen, 2006; Zhang et al., 2017), we chose to explore a maximum of 4 generations with a generation size of 8. So, during the optimization period, participants ran 32 (4x8) times for 90 s on the experimental running shoes with intervening rest periods of 60 s. We calculated the mean positive ankle work of the first 6 successive steps of the dominant leg during the last 30 s of a 90‐s trial. The result was used as input for the optimization algorithm, with a higher value indicating a better outcome. To ensure that the optimal parameters were an actual measured setting, eventually all 32 trials were compared and the apex position and apex angle that resulted in the highest positive ankle work were chosen as optimal parameters. An example of the results of the optimization period of 1 participant is presented in Figure 1C–D. See Appendix for the results of every individual participant.

In the evaluation phase, participants ran 3 times for 6 min in different shoe conditions: (1) experimental shoes with optimal parameter settings, (2) experimental shoes with standard parameter settings (apex position: 64% total shoe length, apex angle: 88.0°; copied by visual inspection of the apex of the control shoe) and (3) control shoes. We measured oxygen consumption and carbon dioxide production to compare running economy. Ground reaction forces and marker trajectories were measured for biomechanical analysis. In between trials, participants took a 5‐min break while they changed shoes. Shoes were worn in random order.

### 2.5 Data analysis

Biomechanical data of the dominant leg in the last 2 min of the evaluation period were used in the offline data analysis. Data were filtered with a low‐pass second order Butterworth filter with cutoff frequency of 6 Hz. We used a custom‐made MATLAB (R2021b, MathWorks, Natick) script with a 10 N vertical ground reaction force threshold to detect the different steps and calculated stance time and step frequency. We calculated the course of vertical ground reaction force and anterior–posterior point of force application over the stance time. For the ankle, knee, and hip joint we calculated joint angles, angular velocities, moments, and powers during the stance phase, using custom MATLAB scripts. Positive and negative joint work was calculated by integrating the power curves over time, for the intervals of positive and negative power, respectively. To gain insight into the contribution of the positive work generated around different joints, we expressed the share as a percentage of the total positive work generated around the lower limb joints. To compare the biomechanical effects of the different shoe conditions, we time normalized all data curves to the duration of the stance phase. The metabolic cost of running was calculated based on the mean $\dot{V}$O_2_ and $\dot{V}$ *CO*_2_ over the last 2 min of each trial, using the equation of Garby and Astrup (Garby et al., 1987):

$$Metabolic\;Power=16.04*\dot{V}O_{2}+4.94*\dot{V}CO_{2}$$

where metabolic power is in W and $\dot{V}$ *O*_2_ and $\dot{V}$ *CO*_2_ are in mL/s. Subsequently, we normalized metabolic power for body mass (W/kg).

### 2.6 Statistical analysis

We performed repeated measures analyses of variance (ANOVA), in SPSS (Version 28.0.1, IBM Corp.), with shoe condition (optimal, standard, control) as within‐subjects factor for 2 dependent variables: positive ankle work and metabolic cost of running. A *p*‐value of 5% was used to indicate statistically significant differences. We used Bonferroni corrected post hoc paired *t*‐tests to compare the different shoe conditions. If the assumption of sphericity was violated, we used a Greenhouse–Geisser correction. Time series of continuous gait variables were compared using 1‐dimensional Statistical Parametric Mapping (SPM‐1D) (Pataky, 2010). All SPM‐1D analyses were performed with a custom MATLAB script and using the open‐source software package spm1D 0.4.8 (www.spm1d.org). SPM‐1D repeated measures ANOVA were used to compare the shoe conditions (*p* < 0.05). The *p*‐value of the post hoc paired *t*‐tests were Bonferroni corrected.

## 3 RESULTS

All optimized apex positions were more distally placed compared to the standard apex position (Table 1). In 8 of the 10 cases, the final apex angle was smaller than the standard angle (88°).

**TABLE 1:** Optimized apex position (as percentage of total shoe length) and apex angle for each participant (*n* = 10).

	Apex position (%)	Apex angle (˚)	Optimal trial (n)	Color	
Optimal parameters					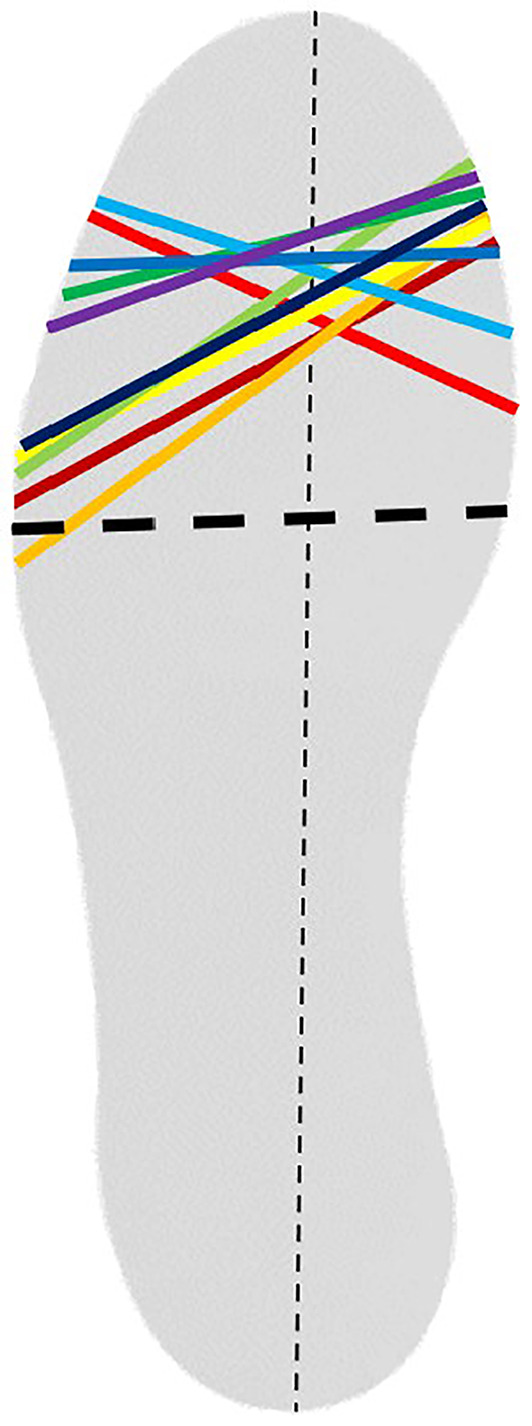
Participant 1	77.2	61.1	18	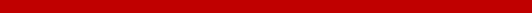	
Participant 2	78.7	115.1	31	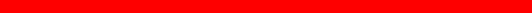	
Participant 3	75.9	53.3	11	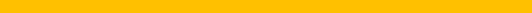	
Participant 4	78.6	60.8	24	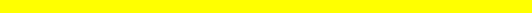	
Participant 5	81.0	54.5	11	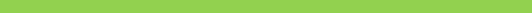	
Participant 6	84.2	76.2	13	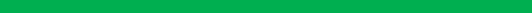	
Participant 7	81.6	107.5	8	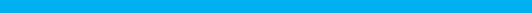	
Participant 8	82.4	87.4	7	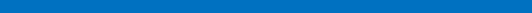	
Participant 9	79.5	62.4	20	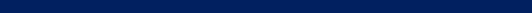	
Participant 10	84.2	66.9	31	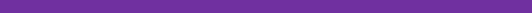	
Standard parameters	64.0	88.0		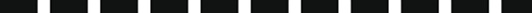	

*Note*: Number of trial during the optimization period (maximum = 32) that resulted in the optimal parameters is also given.

During the evaluation phase, no significant differences were found in step frequency and stance time (Table 2). A significant shoe condition effect was found for positive knee and ankle work and negative ankle work (Table 2). Post hoc tests revealed that running on experimental shoes with optimal apex parameters and control shoes resulted in significantly higher positive work compared to experimental shoes with standard apex settings (+22.4%, *p* < 0.01 and + 29.6%, *p* < 0.01, respectively; Table 2). Significant differences between the optimized settings and the control shoe were not found.

**TABLE 2:** Descriptives (mean (SD)) and results of repeated measure analyses of variance of running parameters, lower limb joint work, and metabolic cost of running for different shoe conditions (*n* = 10).

	Apex optimal		Apex standard		Control		F (2,18)	*p*	ES^a^
Step frequency (step/s)	2.67	(0.07)	2.70	(0.08)	2.70	(0.11)	1.20	0.32	0.12
Stance time (s)	0.313	(0.024)	0.316	(0.022)	0.312	(0.023)	1.96	0.17	0.18
Positive hip work (J/kg)	0.22	(0.08)	0.24	(0.08)	0.23	(0.09)	2.25	0.14	0.20
Positive knee work (J/kg)	0.37	(0.08)^S^	0.42	(0.05)^O,C^	0.35	(0.09)^S^	9.15^b^ ^,^ ^c^	0.01	0.50
Ankle work (J/kg)									
Positive	1.20	(0.27)^S^	0.98	(0.20)^O,C^	1.27	(0.30)^S^	21.06^c^	<0.001	0.70
Negative	−0.44	(0.13)^S^	−0.30	(0.09)^O,C^	−0.42	(0.11)^S^	26.37^c^	<0.001	0.75
Lower limb positive work (% of total)									
Hip	12.09	(3.82)^S^	14.66	(3.62)^O,C^	12.38	(5.02)^S^	8.47^c^	<0.01	0.49
Knee	21.06	(4.26)^S,C^	26.07	(3.17)^O,C^	19.24	(3.97)^O,S^	56.06^b^ ^,^ ^c^	<0.001	0.86
Ankle	66.85	(4.38)^S^	59.26	(4.46)^O,C^	68.38	(5.28)^S^	60.25^c^	<0.001	0.87
Metabolic cost (W/kg)	14.47	(1.81)^C^	14.58	(1.59)^C^	13.88	(1.95)^O,S^	10.12^c^	0.001	0.53

^a^ Effect Size = partial η2.

^b^ Greenhouse–Geisser correction was used.

^c^ Bonferroni corrected post hoc tests (^O^vs. Apex Optimal; ^S^vs. Apex Standard; ^C^vs. Control).

**FIGURE 2:**
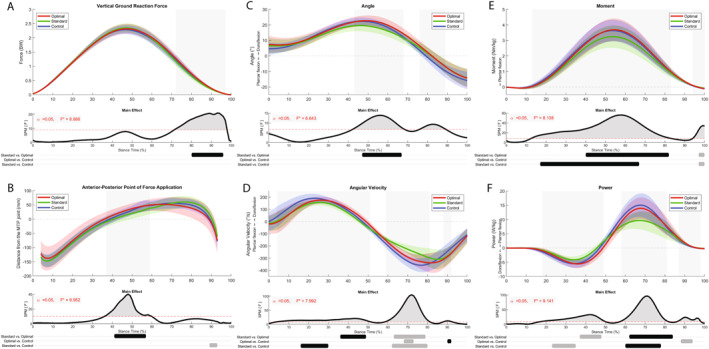
(A) Vertical ground reaction force, (B) anterior–posterior distance between point of force application and metatarsophalangeal (MTP) joint, and sagittal ankle (C) angle, (D) angular velocity, (E) moment, and (F) power (Mean ± SD) patterns for all shoe conditions. The gray areas indicate significant differences (*p* < 0.05). The time dependent F‐values of the SPM‐1D repeated measures ANOVA and results of the post hoc tests between the shoe conditions are given underneath (gray bar = increase; black bar = decrease).

SPM‐1D with repeated measures ANOVA revealed significant differences between shoe conditions in the vertical ground reaction force during 72%–97% of stance time (*p* < 0.001; Figure 2A). A main effect for the anterior–posterior point of force application was found during 37%–59% of stance time (*p* < 0.001). Post hoc test showed that the optimal condition resulted in a significantly more distally placed point of force application during this interval, compared to the standard condition (Figure 2B).

The main effect for the ankle angle was found during 43%–68% and 76%–89% of stance time (Figure 2C). The optimal apex condition resulted in a larger angle (dorsiflexion) compared to the standard apex condition (47%–67% stance time; *p* < 0.01). Angular velocity showed most evident contrasts during 59%–81% of stance time (Figure 2D). Post hoc test revealed a significantly higher plantar flexion angular velocity for the optimal (63%–79% stance time) and control condition (62%–80% stance time) compared to the standard condition.

Ankle moments differed between conditions 13%–83% of stance time (*p* < 0.001; Figure 2E), with a higher plantar flexion moment for the optimal condition (40%–82% stance time) and control condition (17%–67% stance time) compared to the standard condition. The power patterns differed most evident during 18%–49% (*p* < 0.001; Figure 2F) and 58%–81% (*p* < 0.001) of stance time. The post hoc test revealed that power absorption was reduced for the standard condition compared to the optimal condition (37%–48% stance time) and control condition (23%–35% stance time). Significantly, more plantar flexion power is generated during the optimal condition (62%–84% stance time) and control condition (60%–78% stance time) compared with the standard condition.

The shoe conditions altered the share of positive hip, knee, and ankle work as percentage of the total positive work generated around the lower limb joints (Table 2). Post hoc tests revealed that the optimized apex condition resulted in a significantly higher share of positive ankle and lower knee and hip work compared to the standard apex condition. The control shoe also resulted in a higher share of positive ankle work and lower knee work compared to the standard condition.

A significant shoe condition effect was found for metabolic power (Table 2). The conditions wearing the experimental shoes with optimal (*p* = 0.01) and standard (*p* = 0.02) apex parameters resulted in a higher metabolic cost of running compared to the control shoe. A significant difference between the optimal condition and standard condition was not found.

## 4 DISCUSSION

The purpose of the present study was to determine if human‐in‐the‐loop optimization could optimize apex position and angle to maximize positive ankle work and whether this would increase running economy. We hypothesized that this would be achieved by a more distally placed apex position and an apex angle close to 90°. We expected that this would result in a higher percentage of positive ankle work with respect to total lower limb positive work and a lower metabolic cost of running. For all participants, the optimal apex position was placed more distally compared to the standard position. The optimal apex angles showed more variation between participants and were not centered on the expected 90°. Consistent with our hypothesis, the human‐in‐the‐loop optimization resulted in higher positive ankle work and redistribution of joint work compared to the standard settings. However, this did not result in a difference in running economy between the optimal and standard settings.

Our findings show that the optimal condition, with a more distally placed apex position, results in a faster distal travel of the point of force application compared to the standard condition. This increases the moment arm of the ground reaction force with respect to the ankle joint, which requires a higher internal ankle moment. To initiate foot rotation, the internal ankle moment generated by the ankle plantar flexor muscles should exceed the external ankle moment from the ground reaction force (Houdijk et al., 2003). The higher external moment due to the larger moment arm of the ground reaction force in the optimal condition delays the start of foot rotation, which is confirmed by an increase in peak dorsiflexion. The increased ankle moment and increased range of motion of the ankle (and concomitant higher angular velocity) result in an increase of both negative and positive ankle power and work during the optimal condition compared to the standard condition and could explain the corresponding redistribution of joint work from the hip and knee to the ankle.

We expected this would result in an improved running economy compared to the standard condition, because due to the increased force on the *m*. Triceps Surae more negative work could be stored in the tendon of the Triceps Surae (Ishikawa et al., 2007; Roberts, 2002), and subsequently more stored energy could be reused during the propulsion phase. In addition, muscle fiber shortening could be reduced because the tendon can take up more of the muscle–tendon unit length change. This could limit muscle fiber shortening velocities and enhance muscle mechanical efficiency (Hill, 1938).

However, our results show no difference in metabolic cost between the optimal and standard conditions. Unfortunately, the current study design does not allow to assess the muscle–tendon contraction behavior and to test the mechanism described above. A possible explanation for the lack of effect in running economy between optimized and standard shoe condition could be the cost of more force production in the Triceps Surae muscle (Griffin et al., 2003) due to the increased peak dorsiflexion moment (Miller et al., 2014). Moreover, the ankle range of motion was increased during the optimal condition, causing an increased change in muscle–tendon unit length (Bohm et al., 2019). In addition, the higher force might have caused more stretch in the tendon unit at a given range of motion, increasing the shortening of the muscle fibers. Because of this, it could be that muscle fiber shortening velocities were less limited than expected and the hypothesized increase in mechanical efficiency did not occur.

Comparing the optimal condition with the control condition, that is, a regular athletic shoe, we see that the running biomechanics show many similarities. Although the control shoe had a more proximal apex position, differences in anterior–posterior point of force application were not found. A possible explanation is that due to the lower bending stiffness of the control shoe, intrinsic foot muscles were able to manipulate this position (Miller et al., 2014) to the individual's optimum. This is in line with previous research (Fuller et al., 2016), which demonstrated that running in minimalist shoes resulted in increased negative and positive ankle work and decreased knee work, showing the same redistribution of joint work.

Despite the biomechanical similarities between the optimal and control conditions, there was a difference in running economy. A plausible explanation for this finding could be an increased lower limb moment of inertia (Moore, 2016) due to the extra mass of the experimental shoes. Previous studies (Franz et al., 2012; Hoogkamer et al., 2016) found an increase in the oxygen cost of approximately 1% per 100 g of extra shoe mass per foot, where Rodrigo‐Carranza et al. (2020) (Rodrigo‐Carranza et al., 2020) found even a higher increment when running economy was expressed as energy cost instead of oxygen cost only. Given the fact that the mass of the experimental shoes was over 200 g higher per foot than the control shoe, explains the difference in running economy of 4% to a great extent.

### 4.1 Evaluation human‐in‐the‐loop optimization

The fact that the optimal apex position ranged from 75% to 84% and the optimal apex angles showed even more variance (range: 55–115°) emphasizes the importance of this individual approach. Our results demonstrate that human‐in‐the‐loop optimization is able to find optimal parameter values that are beyond the range that is used in regular practice and would not have been found during grid search based on apex parameter values used in existing literature (Chapman et al., 2013).

Another challenge this method is able to deal with is the fact that humans exhibit individualized adaptation and learning processes (Zhang et al., 2017). This embedded tuning of motor control could contribute substantially to the final effect (Poggensee et al., 2021). After every generation, the estimate of the optimal parameter values is updated, and the area of search around this estimate is further decreased or increased (Hansen, 2006) to explore potentially better parameter settings. Because of this, the effect of motor learning during the optimization process maximally contributes to the measured positive ankle work when the optimal apex parameters are approached, which prevents potentially beneficial apex parameters with early, poorly adapted motor skills to be overlooked (Zhang et al., 2017). Moreover, the characteristic of human‐in‐the‐loop optimization of variation in practice, followed by more focused training near the optimal solution have been shown to result in faster adaptations compared to training without variation (Poggensee et al., 2021).

Nevertheless, the current results showed that parameter values during the final generation still show relatively high variance and convergence to the optimal setting did not always occur. We used a relatively large generation size of 8 to improve global search capability and robustness of the algorithm (Hansen, 2006). However, a consequence of this decision is reduced convergence speed (Hansen, 2006). It could be that this decision did not match with the chosen initial standard deviation and therefore this value did not decrease sufficiently toward the final generation. This could have caused that the optimized apex parameters may not have been reached after the maximum of 4 generations, and could explain why only 2 participants had their optimal trial in the final generation.

### 4.2 Limitations and future research

One important note that has to be made is that some participants seemed to run with a high vertical displacement, which resulted in higher amplitudes in the vertical ground reaction force and higher peak moments than previous studies with low running speeds reported (Sobhani et al., 2013). This higher vertical ground reaction force and ankle moments coincided with shorter stance time, longer flight time, and higher vertical pelvis displacement, but we could not find specific explanations for this behavior. The fact that only 2 participants had their optimal trial in the final generation of the optimization period supports the idea that participants were sufficiently adapted to the shoes and contradicts the idea that this might be an effect of adaptation. Moreover, this atypical running pattern was also seen in the condition with the control shoe and thus not a negative side effect of the experimental shoes. A possible explanation for this result might be the fact that participants wore a portable spirometer on their back during data collection and this load carriage induced the atypical running pattern (Wang et al., 2023).

Although the generalizability should be considered with caution, the study showed that human‐in‐the‐loop optimization is able to optimize the selected objective, that is, maximize positive ankle work, and we believe that this method has the potential to individually boost the effectiveness of footwear and assistive devices. However, it did not result in the anticipated increase in running economy. The relation between ankle work and gait economy should be further explored. Moreover, it could be questioned whether ankle work is the most effective cost function for optimizing running shoes (Sobhani et al., 2013).

By means of the human‐in‐the‐loop optimization method, the adjustable parameters can be tuned to biomechanical properties of the individual (Scotto di Luzio et al., 2018), while taking into account the natural behavior of humans to simultaneously optimize coordination patterns with respect to the locomotor performance (Alexander, 1989). However, the current design does not reach the methods' full potential. Therefore, future research should investigate whether the current genetic optimization algorithm could be improved or test other potential optimization algorithms to improve the convergence rate of the optimization process, and/or the magnitude of the final effect. Furthermore, it should be investigated how optimization via different cost functions (e.g., physiological, biomechanical, and balance) influences human movement in different settings. If we could get a grip on these questions, we will be better able to individually adjust footwear or other assistive devices to the desires of the user.

## 5 CONCLUSION

The present study shows that human‐in‐the‐loop optimization is able to enhance positive ankle work during running by individually optimizing apex position and apex angle. Although we found an increase in ankle work and redistribution of joint work from the hip and knee to the ankle, the expected improvement in running economy was not found. It needs to be further investigated which factors confound the theoretical benefits of enhanced ankle work on running economy. Furthermore, human‐in‐the‐loop algorithms and cost functions could be further improved to better converge to optimal individual solutions.

## AUTHOR CONTRIBUTIONS

Thijs Tankink conceptualized the study, collected, processed, and analyzed the data, interpreted the results, and wrote the initial draft of the manuscript; Han Houdijk conceptualized the study, analyzed the data, interpreted the results, and revised the manuscript; Juha M. Hijmans conceptualized the study, analyzed the data, interpreted the results, and revised the manuscript. All authors have read and approved the final version of the manuscript and agree with the order of presentation of the authors.

## CONFLICT OF INTEREST STATEMENT

The authors declare that they have no competing interests.

## Supporting information

Supplementary Material
