# Supplementary material for: Human‐in‐the‐loop optimized rocker profile of running shoes to enhance ankle work and running economy
Source: Eur J Sport Sci. 2024 Jan 30;24(1):164–73. doi: 10.1002/ejsc.12054 (PMC11235892; doi:10.1002/ejsc.12054)

**Appendix**

Results of the iterations in the optimization period for every participant. For every trail, the cylinder positions (medial and lateral; as percentage of the total shoe length) and corresponding positive ankle work (vertical axis) are displayed. The small figure in the right bottom corner shows the used cylinder positions (as percentage of the total shoe length) of every trial in a 2D top view. The optimal trial is represented by the star. The different colors indicate the different generations.


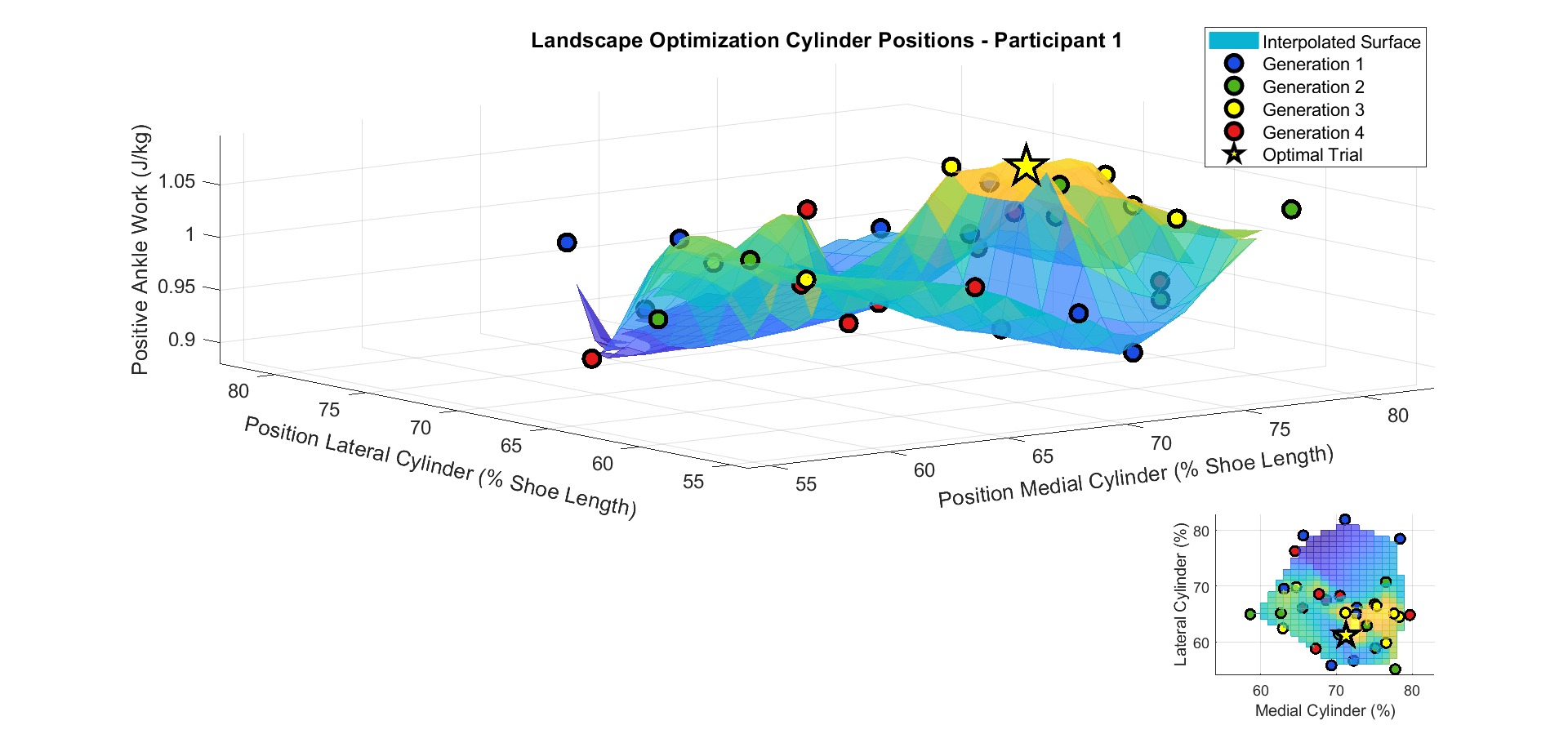


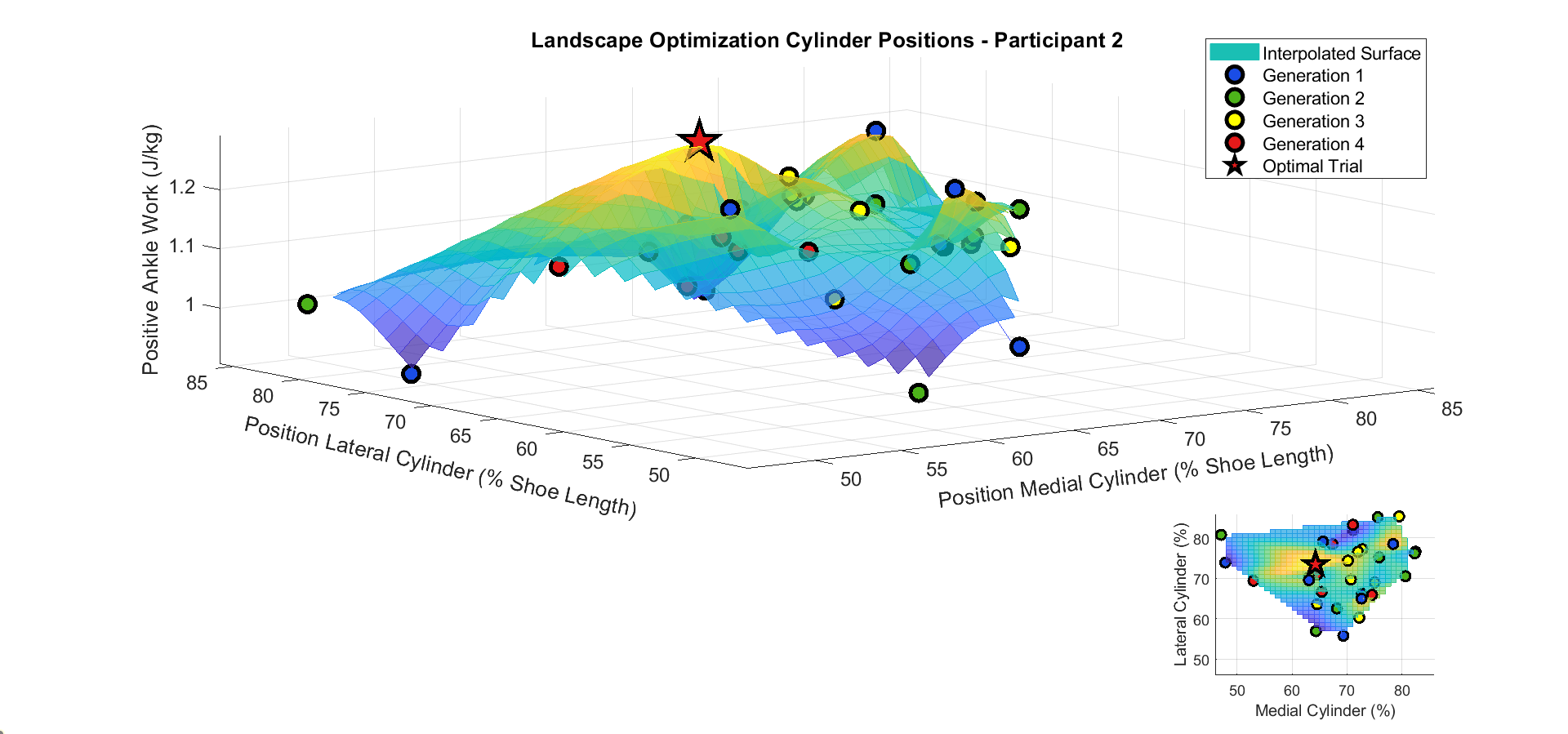


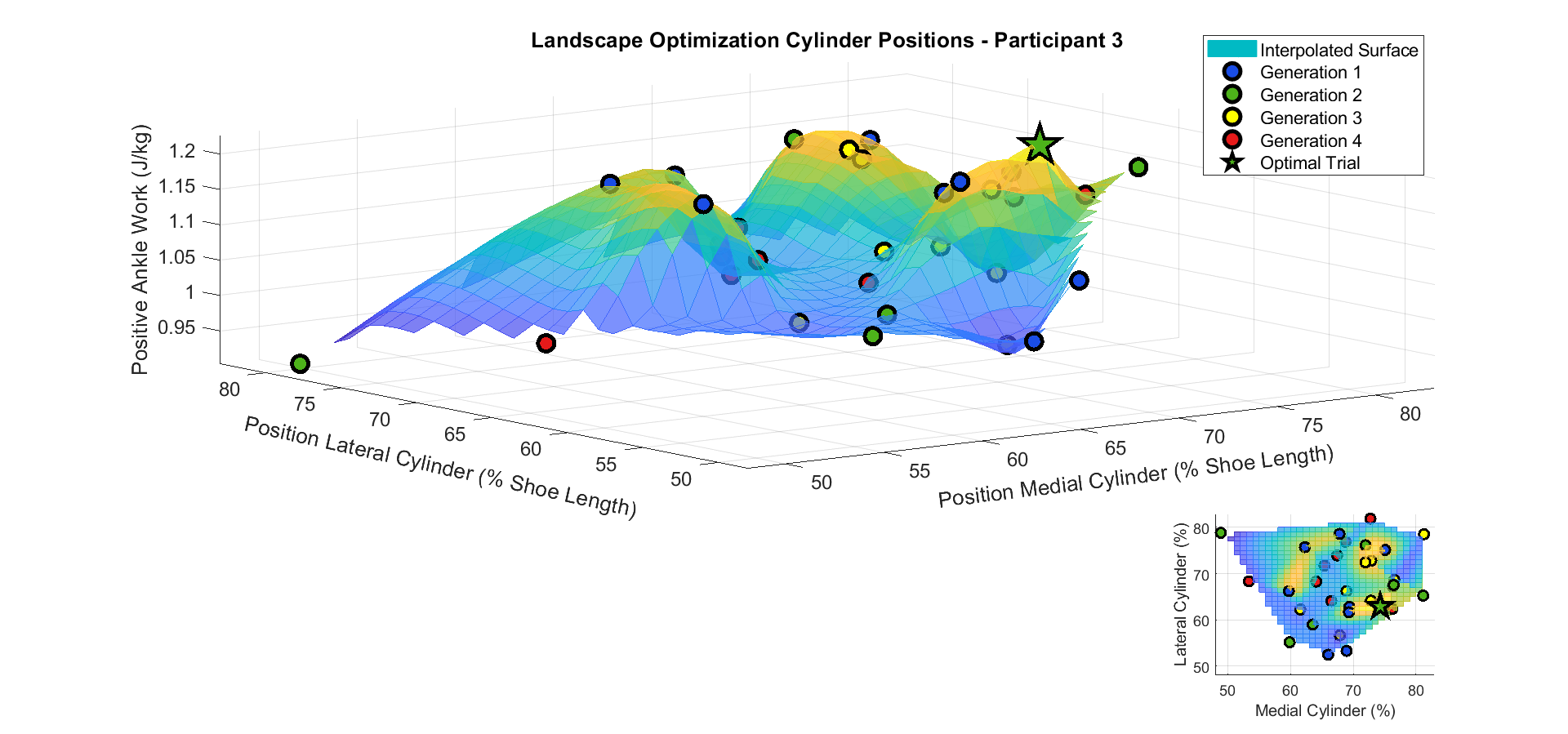


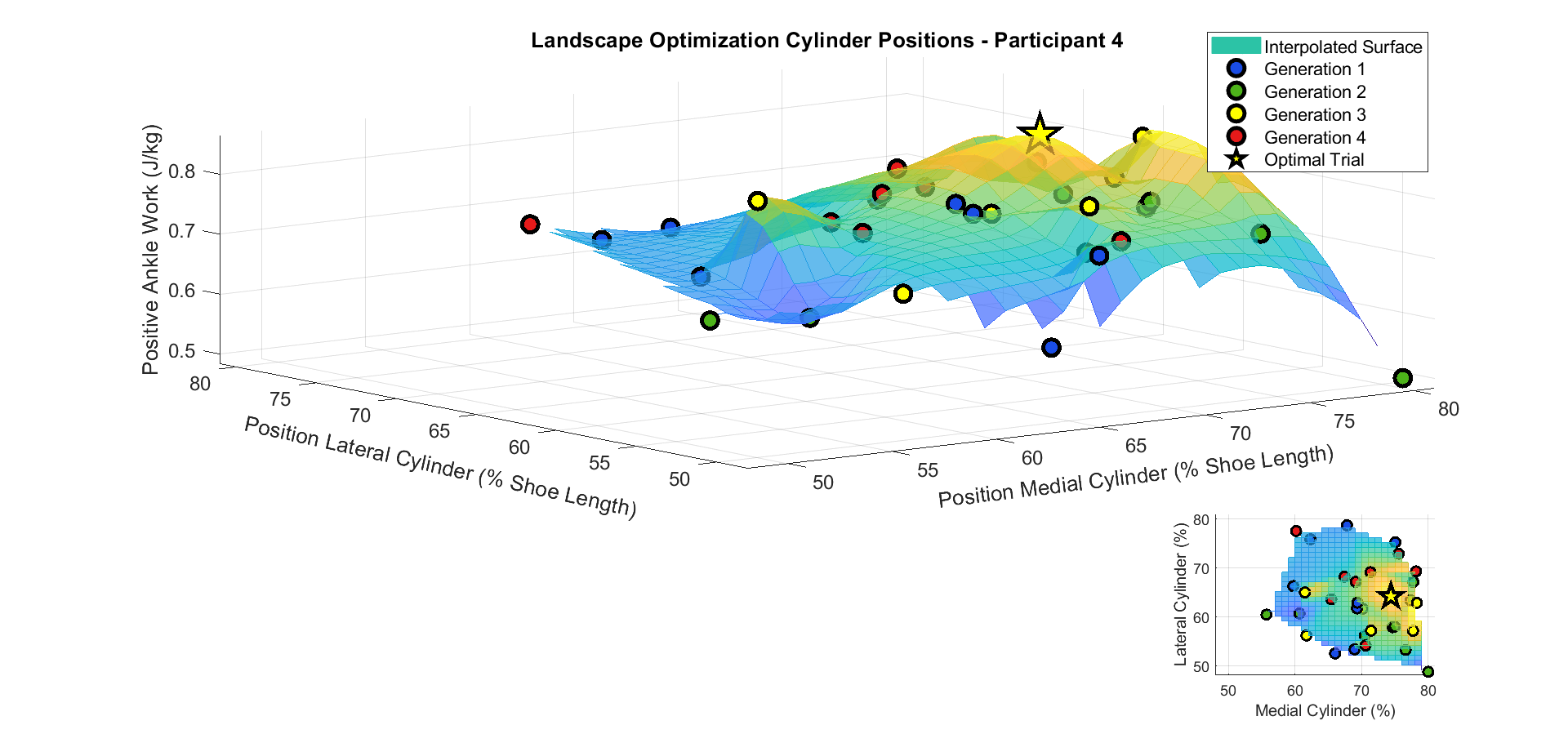


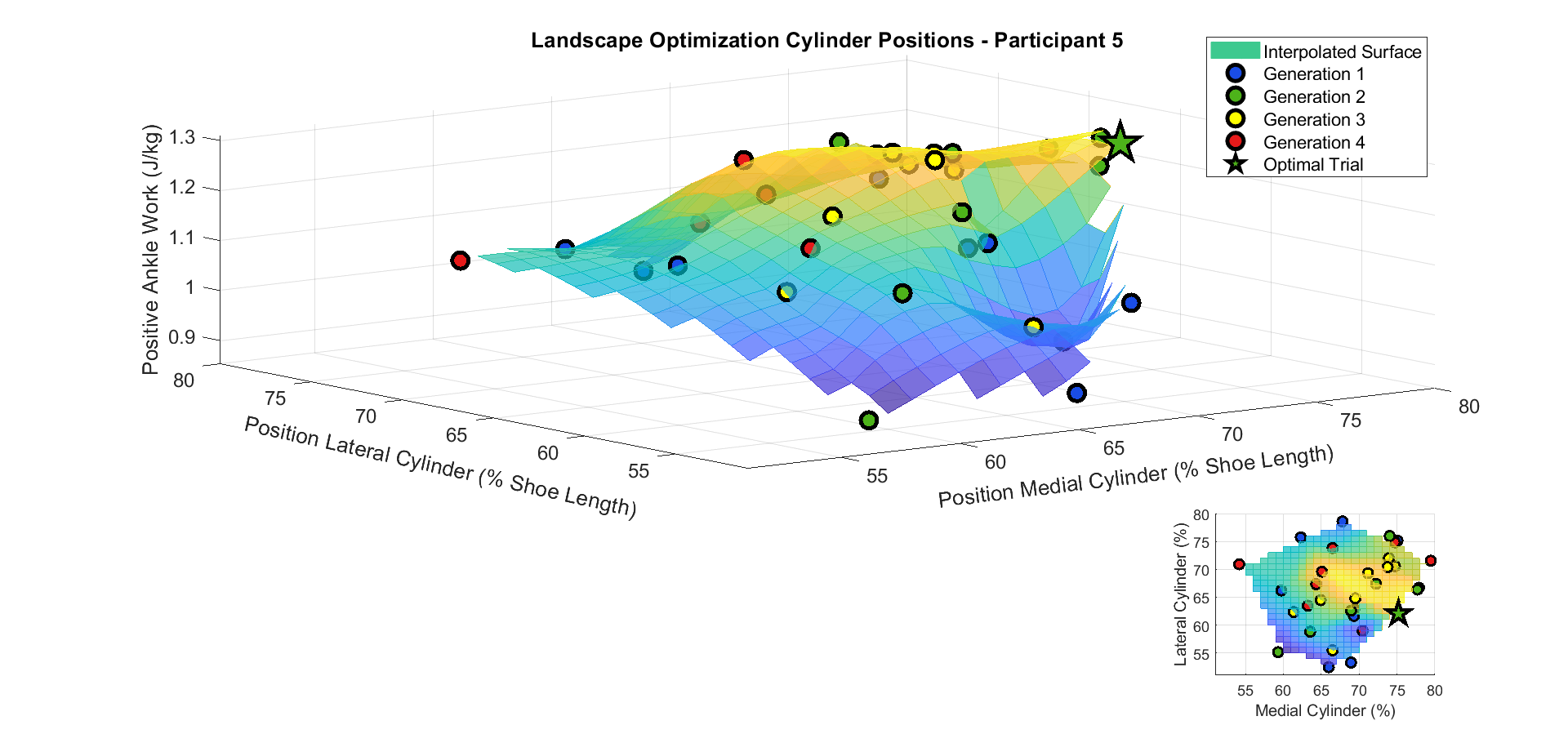


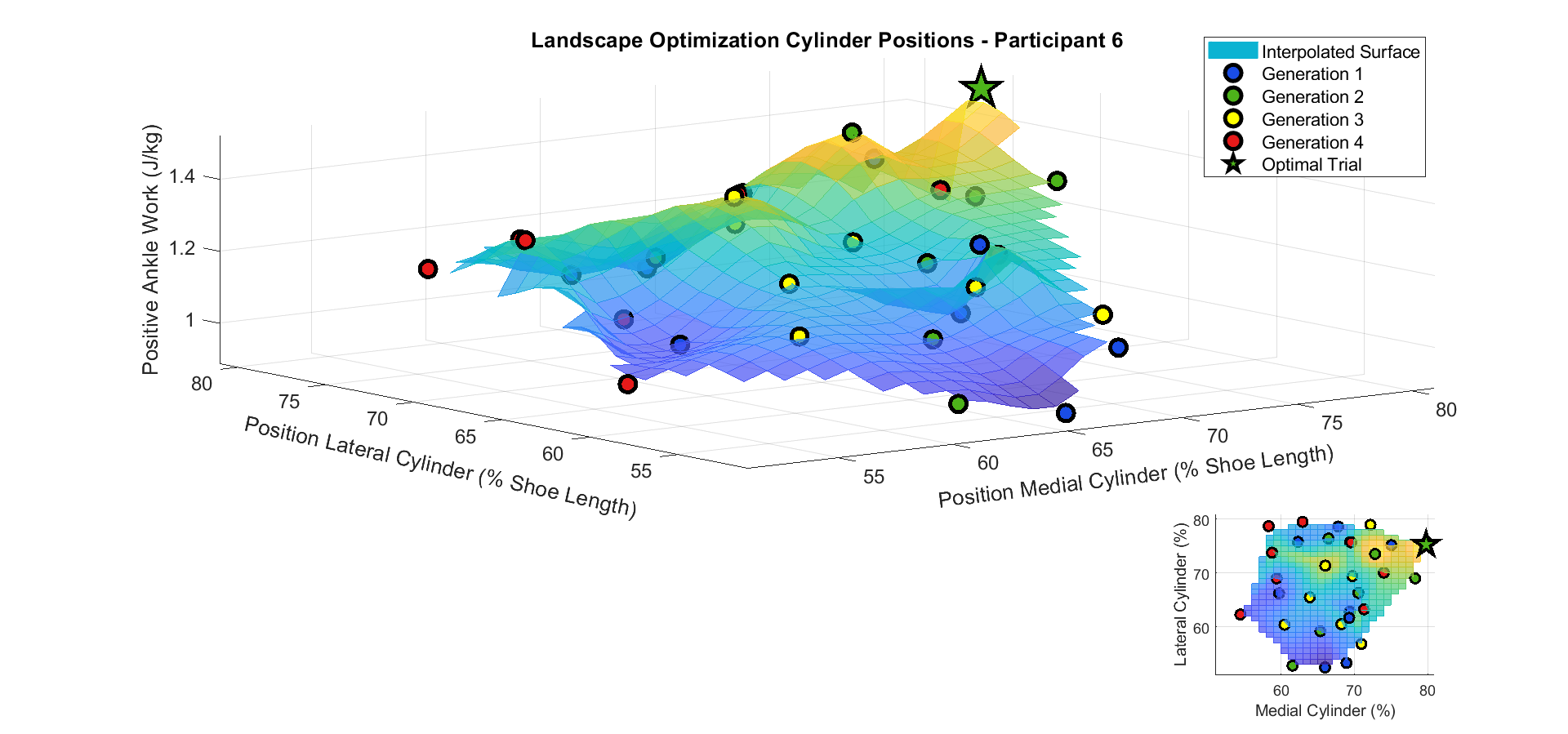


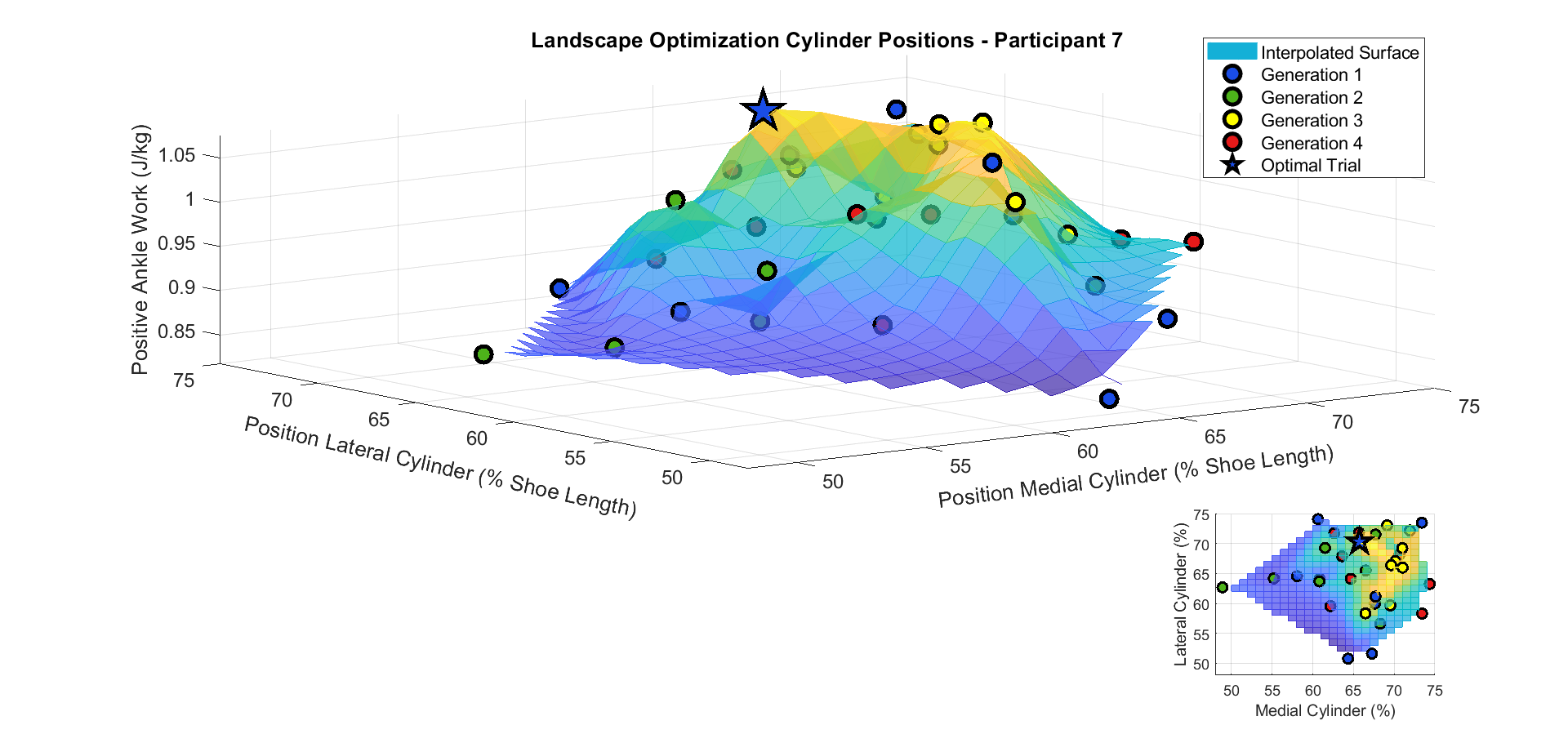


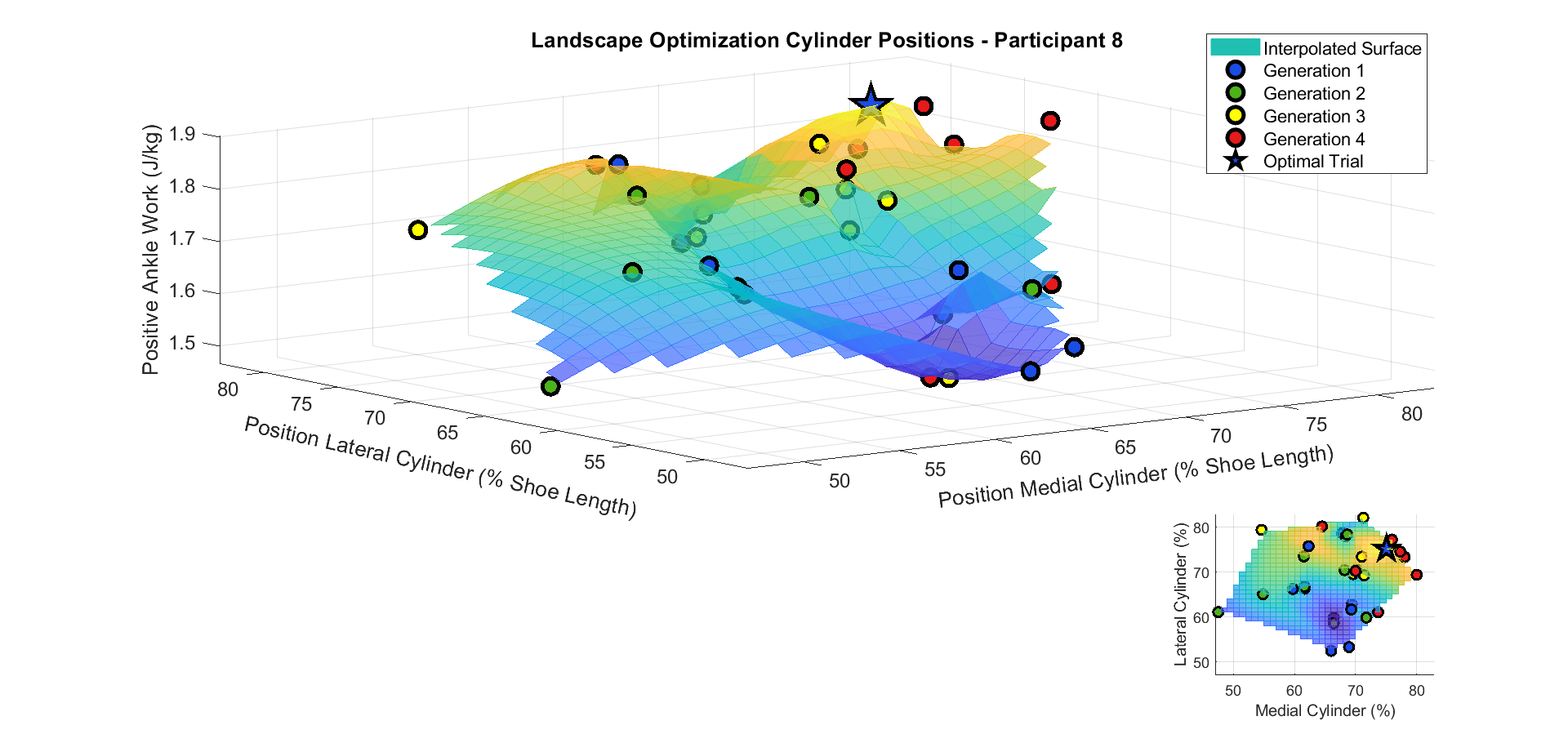


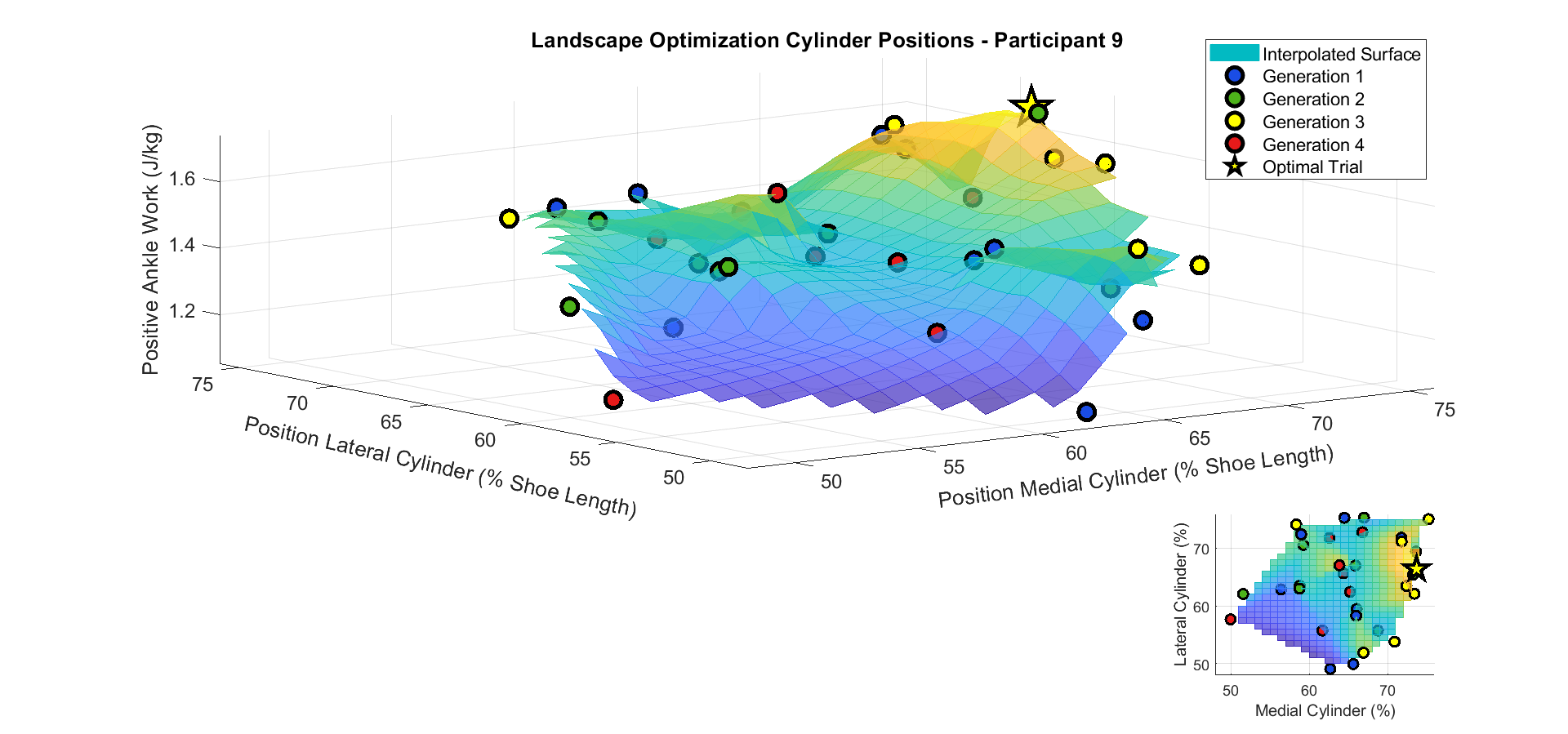


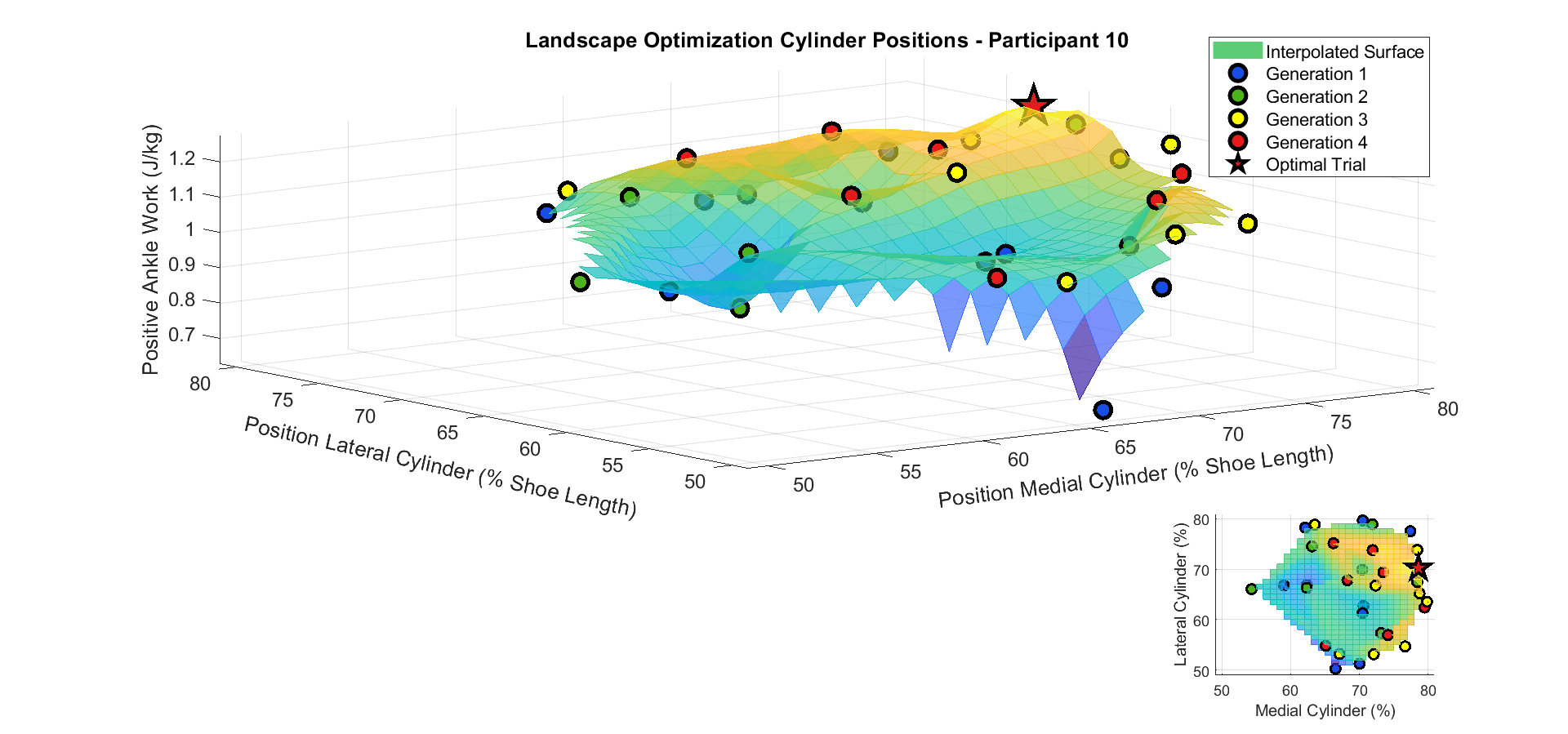

Supplement: Supplementary file 1 — Supplementary Material [file EJSC-24-164-s001.docx]
